# Childhood adversity and self-poisoning: A hospital case control study in Sri Lanka

**DOI:** 10.1371/journal.pone.0242437

**Published:** 2020-11-19

**Authors:** Thilini Rajapakse, Abigail Emma Russell, Judi Kidger, Piumee Bandara, José A. López-López, Lalith Senarathna, Chris Metcalfe, David Gunnell, Duleeka Knipe

**Affiliations:** 1 Department of Psychiatry, Faculty of Medicine, University of Peradeniya, Peradeniya, Sri Lanka; 2 Centre for Academic Mental Health, Population Health Sciences, University of Bristol Medical School, Bristol, United Kingdom; 3 Population Health Sciences, Bristol Medical School, University of Bristol, Bristol, United Kingdom; 4 Translational Health Research Institute, Western Sydney University, Sydney, New South Wales, Australia; 5 Department of Basic Psychology & Methodology, Faculty of Psychology, University of Murcia, Murcia, Spain; 6 Department of Health Promotion, Faculty of Applied Sciences, Rajarata University of Sri Lanka, Mihinthale, Sri Lanka; 7 National Institute of Health Research Biomedical Research Centre at the University Hospitals Bristol NHS Foundation Trust and the University of Bristol, Bristol, United Kingdom; 8 The South Asian Clinical Toxicology Research Collaboration (SACTRC), Faculty of Medicine, University of Peradeniya, Peradeniya, Sri Lanka; Harvard Medical School, UNITED STATES

## Abstract

**Introduction:**

Adverse childhood experiences (ACE) have been recognized as an important risk factor for suicidal behaviour among adults, but evidence from low and middle-income countries is lacking. This study explored associations between ACE and hospital admission due to non-fatal self-poisoning in Sri Lanka.

**Methods:**

This was a case-control study. Adults admitted to a tertiary care hospital for medical management of self-poisoning were included as cases, and age and sex matched controls were recruited from the outpatient department. ACE were measured using the World Health Organization’s Childhood Adversity Scale. Logistic regression models adjusting for age, sex, ethnicity, and religion were used to quantify the association between ACE and self-poisoning.

**Results:**

The study included 235 cases and 451 controls. Cases were 2.5 times (95% CI 1.8, 3.6) more likely to report an ACE than controls and had higher ACE scores. Childhood physical abuse (OR 4.7, 95% CI 1.2, 19.0) and emotional abuse or neglect (OR 3.7, 95% CI 1.3, 10.1, and 3.7, 95% CI 2.3, 6.0 respectively), increased the risk of self-poisoning in adulthood, as did witnessing household violence (OR 2.2, 95% CI 1.4, 3.4), growing up in a household with a mentally ill or suicidal household member (OR 2.1, 95% CI 1.2, 3.4), and experiencing parental death/separation/divorce (OR 3.1, 95% CI 2.0, 4.9) as a child.

**Conclusions:**

Reducing exposures to ACEs should be a priority for prevention of suicide and self-harm in Sri Lanka. Innovative methods to increase support for children facing adversity should be explored.

## Introduction

Suicide is a significant cause of mortality, with 800,000 deaths reported globally each year due to suicide [1]. For every suicide death, an estimated 20 people self-harm [2]. Understanding the factors that contribute to increased risk of suicide and suicidal behaviour at an individual level is important in order to design effective interventions.

Adverse exposures in early life have long been recognized as important risk factors for suicidal behaviour [3]. Childhood adversity includes exposure to physical, emotional or sexual abuse, as well as experience of emotional and physical neglect, and witnessing of domestic violence as a child [4]. Childhood adversity is a key risk factor for suicide in high income countries (HIC), but evidence from low and middle income countries (LMIC) is limited [3, 5–7]. Studies exploring this association in South Asia are minimal. Studies specifically investigating the association between childhood adversity and suicidal behaviour in LMIC and South Asia are needed. This is important as children growing up in LMIC are more likely than those from HIC to experience childhood adversity such as poverty, parental death, parental alcohol misuse and associated domestic violence [8]. Research is therefore needed to explore whether childhood adversity is a strong risk factor for suicide in LMIC, in order to understand whether prevention efforts developed in HIC are likely to be generalizable across cultures.

Sri Lanka historically had a very high rate of suicide, but with the introduction of a series of national pesticide bans, the death rate due to suicide has declined substantially [9, 10]. Despite the reduction in the number of suicide deaths, there has been a concurrent rise in non-fatal self-poisoning by medicinal products, primarily by adolescents and young adults [11]. Sri Lanka also suffered from a protracted civil war that lasted 26 years (1983–2009) that resulted in a large number of deaths. Children growing up during this time are likely to have experienced high levels of bereavement and community violence. Sri Lanka also has a high rate of alcohol consumption, with associated domestic violence, and consequently many children grow up in households with domestic violence [12–14].

### Aims of the study

To the best of our knowledge, the relationship between childhood adversity and self-harm in Sri Lanka has not been previously investigated. Using data collected as part of a hospital-based case control study, we aimed to explore the association between exposure to childhood adversity (including type of adversity e.g. physical abuse/ experiencing violence in the home) and hospital presenting non-fatal self-poisoning in Sri Lanka. We hypothesised that exposure to childhood adversity would be associated with increased risk of hospital admission for non-fatal self-poisoning.

## Methods

### Study setting

This was a hospital-based case-control study conducted in the Central Province of Sri Lanka. The study is based at Teaching Hospital Peradeniya (THP), a tertiary care hospital situated in the Central Province of Sri Lanka. The majority of patients admitted to THP are from the same province, but the hospital also receives referrals for tertiary care from the surrounding Provinces.

The study protocol has been previously published [15].

### Study participants

#### Case

Individuals aged 18 years and over admitted to THP for medical management of non-fatal self-poisoning, during July-December 2018, were invited to participate in the study. Patients who were judged by the research team to be physically unable or too unwell to participate prior to discharge from the hospital (those who were unconscious or had a lowered level of consciousness at the time of transfer to another hospital), and those who had previously received a diagnosis from a clinician as being cognitively impaired, were excluded from the study. In cases of uncertainty, the data collectors discussed the issue of study inclusion with the investigators (PB or TR) who referred to the inclusion/exclusion protocol in the decision-making process [15]. Cases of self-poisoning were initially identified from the patient admission record and verbally reconfirmed by the patient through self-report. The degree of suicidal intent was not recorded as part of the interview schedule due to time constraints.

#### Controls

*Hospital based controls*. Sex and age frequency matched controls were recruited from outpatients and accompanying visitors presenting to the THP outpatient department (OPD) and specialist clinics in the same premises between July 2018 to January 2019. Patients present to the OPD for a variety of medical and surgical complaints, such as hypertension, chest infections or cough, which are largely unrelated to the outcome of interest.

*Controls from the community*. The potential for selection bias within the hospital-based control group was identified as a limitation in the study protocol [15]. To address this, an additional control group was recruited from the community. Controls were recruited from the general population corresponding to the population catchment of cases between January 2019 to April 2019. Two administrative divisions where most cases resided were selected for sampling. Within each of the administrative divisions, all Grama Niladhari (GN) sub-divisions (‘villages’) were stratified by sex, age and ethnicity using population census data, and a list of GN subdivisions that were most demographically similar to the wider administrative division was created. All households within the 12 selected GN subdivisions were initially included in the study, but due to difficulties in access, such as hilly terrain, all households within the each subdivision could not be reached. Within each recruited household, the resident who most closely matched the cases according to age (plus or minus five years) and sex, was invited to participate in the interview.

As with cases, individuals who were judged by the research team to be physically unable or too unwell to participate in the study, or those who had previously been diagnosed as being cognitively impaired were excluded as either hospital or community controls. Controls with a self-reported previous self-harm episode were excluded in the analysis.

#### Procedure

All participants took part in an interview, lasting 30–40 minutes. The interview was conducted by a trained interviewer, and was made up of a structured pretested questionnaire in the local language (Sinhala, Tamil or English), and several validated tools (described below). The trained interviewers were recently qualified graduates, with either a basic sciences or nursing degree. In order to reduce bias, interviewers (n = 4) were given a standard script to follow, with regular checks conducted by the supervisor (PB). Further details of the data collection can be found in the protocol paper but are outlined here in brief [15].

Adverse childhood experience (ACE- the main exposure) was measured using the World Health Organisation’s Childhood Experiences International Questionnaire (ACE-IQ) [16–18]. This scale is intended to measure ACEs in all countries and is designed for administration to people aged 18 years and older. Questions cover family dysfunction; physical, sexual and emotional abuse and neglect by parents or caregivers; peer violence; witnessing community violence, and exposure to collective violence. The scale was translated into the local languages (Sinhala and Tamil), and back translated to English, by science or nursing graduates. The scale was then piloted among a small group of outpatients at Teaching Hospital Peradeniya. Any difficulties noted in using the translated scale were discussed by the research team (including PB and TR) and minor adaptations were made where needed. In this study the Cronbach’s alpha for the ACE-IQ was 0.71, indicating acceptable internal reliability.

We also collected data on known confounding factors (age, sex, religion and ethnicity) and a range of potentially confounding/mediating variables. These included the following information, collected via an interviewer administered questionnaire: knowing someone who had previously self-harmed; having a young child (≤11 year old); family size; marital status; occupation; education; asset (vehicle) ownership; and being chronically ill. In addition, we collected data on current depression, using the locally validated Patient Health Questionaire-9 (PHQ9)); the PHQ-9 is an internationally validated, 10-item scale for the screening of depression, which has been translated and validated for use in Sinhala, with a sensitivity and specificity of 0.75 and 0.97 respectively (at a cut off of ≥10) [19, 20]. We screened for alcohol use disorders (using the locally validated Alcohol Use Disorders Identification Test (AUDIT)) and social capital (using questions from a large social capital community survey in Sri Lanka) [21, 22]. The AUDIT is a ten-item questionnaire which has been validated for use in Sinhala in Sri Lanka, and the area under the ROC curve to differentiate hazardous use and alcohol use disorder from low risk drinking is 0.96 [21]. The questions on social capital asked about the individual’s current interpersonal relationships with, and sense of support from, their immediate household and neighbourhood. We explored exposure to domestic violence in the previous 12-months using the four-item Humiliation, Afraid, Rape, Kick (HARK) questionnaire; it explores experience of four types of abuse–physical, sexual, emotional abuse and fear of an intimate partner. For the purpose of this study, the questionnaire was broadened to include abuse by any household member, not just an intimate partner. At an optimal cut-off mark of ≥1, the HARK has a sensitivity of 81% and specificity of 95% [23]. This tool was translated, back translated and piloted in the same manner as described for WHO Childhood Adversity Scale above. The HARK did not require modification after piloting.

#### Ethics

Ethical approval for the study was granted by the Ethical Review Committee of the Faculty of Medicine, University of Peradeniya, Sri Lanka. All data collectors received detailed training in the ethical aspects of the study. All participants were given a verbal explanation of the study with a written information sheet, with a clear explanation that declining to participate would have no impact on medical care. All participants gave written informed consent. All interviews were conducted confidentially in a separate area or room. All data were stored confidentially and in a de-identified manner. Full details of the ethical and safeguarding protocols related to this study have been previously published [15].

#### Analysis

The primary analysis included cases and hospital controls with no missing data. Hospital controls were considered in preference to community controls for purpose of analysis, because the hospital controls more closely matched the residential areas of all cases. Control participants who reported a history of self-harm were excluded (n = 26, 5% of hospital controls).

Following the WHO guidance for calculating an ACE score, we calculated two scores [24]. Each question in the ACE-IQ assesses an ACE. The frequency method counted an individual as having been exposed to a certain ACE if they met a certain frequency threshold (e.g. experiencing the adversity “many times”) whereas the binary method counted individuals regardless of severity (i.e., a simple presence or absence of each ACE). The ACE IQ records frequency of each ACE on a 4-point scale: never; once; a few times; many times. For our frequency analysis we summed the number of ACEs each individual had experienced “many times” (giving a score 0–13). For the binary analysis, participants score 1 if they have experienced each ACE (regardless of frequency), and the total again ranges from 0–13. We used the frequency score in the primary analysis and present the binary score (based on the sum of exposure to each ACE, regardless of frequency) as supplementary analysis. In addition to the continuous score, we also dichotomised the frequency score to indicate presence (continuous score>0) or absence (continuous score = 0) of exposure. We extended this to further explore associations by the type of ACE.

The ACE-IQ questions have been grouped into 13 categories of ACEs. We therefore created 13 categorical variables to indicate presence of one of the ACE sub-categories based on the frequency scoring method: emotional abuse; physical abuse; sexual abuse; violence against household members; living with household members who were substance abusers; living with household members who were mentally ill or suicidal; living with household members who were imprisoned; parental death, separation or divorce; emotional neglect; physical neglect; bullying; community violence; collective violence.

Individuals were categorised as experiencing domestic violence if they answered positively to any of the HARK questions [23]. Based on the local validation of the PHQ-9, a score of 10 or more on the PHQ-9 was used to indicate the presence of depression, and AUDIT scores of 8 or more indicated hazardous drinking behaviour [20, 21]. A continuous score was created for social capital, with higher scores indicating lower social capital.

**Fig 1:**
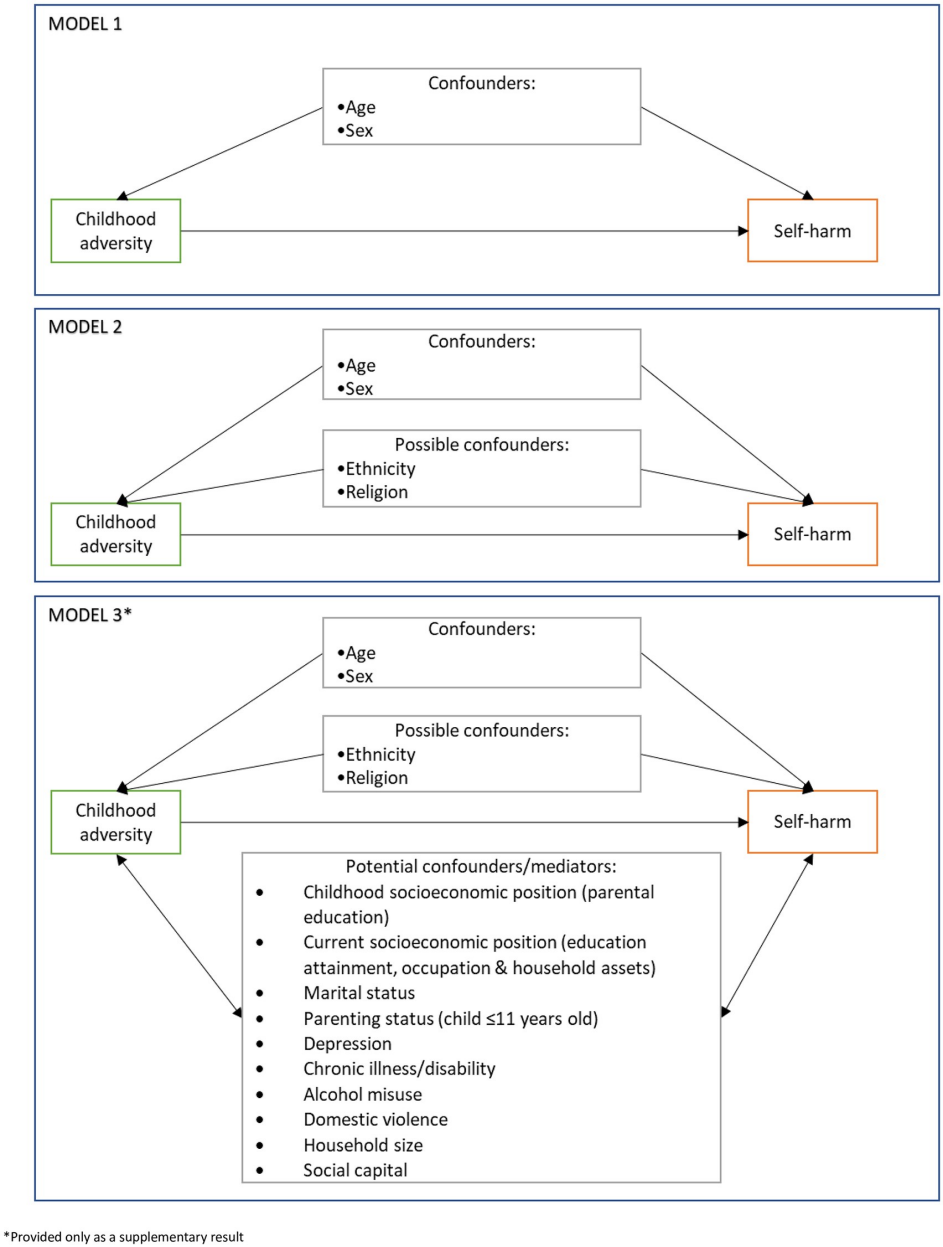
Graphical presentation of the conceptual models used to inform statistical analysis.

Descriptive statistics for the study variables were generated. All variables were coded with the hypothesised lowest-risk category as the reference group. Bivariate relationships between the study variables and the outcome of self-poisoning were assessed using logistic regression models, adjusted for age and sex. The relationship between ACE and self-poisoning was evaluated using a series of logistic regression models (Fig 1). Participant age and sex were included as covariates in all models. We explored the relationship between ACE and self-poisoning adjusting for age and sex only (model 1). We then adjusted for the key confounders of ethnicity and religion (model 2), and this model was used for our main assessment of the association between ACEs and self-poisoning. As an additional analysis we explored the relationship between ACE and self-poisoning adjusting for age, sex, ethnicity, religion and all potentially confounding/mediating variables (model 3). We present this as supplementary results as many of the variables could be mediating variables. The inclusion of mediating variables in our adjusted models can inappropriately suggest an association when one does not exist or give a biased estimate due to over adjustment. In the original protocol we indicated that childhood socioeconomic position was being considered as a confounder, but on reflection we felt that this was unlikely, and that it was more likely to capture aspects of the early environment akin to our other measures of childhood adversity. Therefore, we included childhood socioeconomic position (parental education) in model 3. We also present the confounder adjusted model (model 2) stratified by sex for the overall ACE score, and subcategories. We formally tested to see whether sex modified associations observed for the overall ACE frequency score and presence of any ACE.

We also conducted a series of sensitivity analyses to explore whether our results were robust. We have indicated which of these were defined *a priori*. First, models were repeated using all available data (i.e., including participants with missing data–*a priori* analysis). Second, analyses were repeated using the ACE binary score as the exposure. Finally, we explored associations with the community control group as opposed to hospital controls.

## Results

### Descriptive statistics

**Fig 2:**
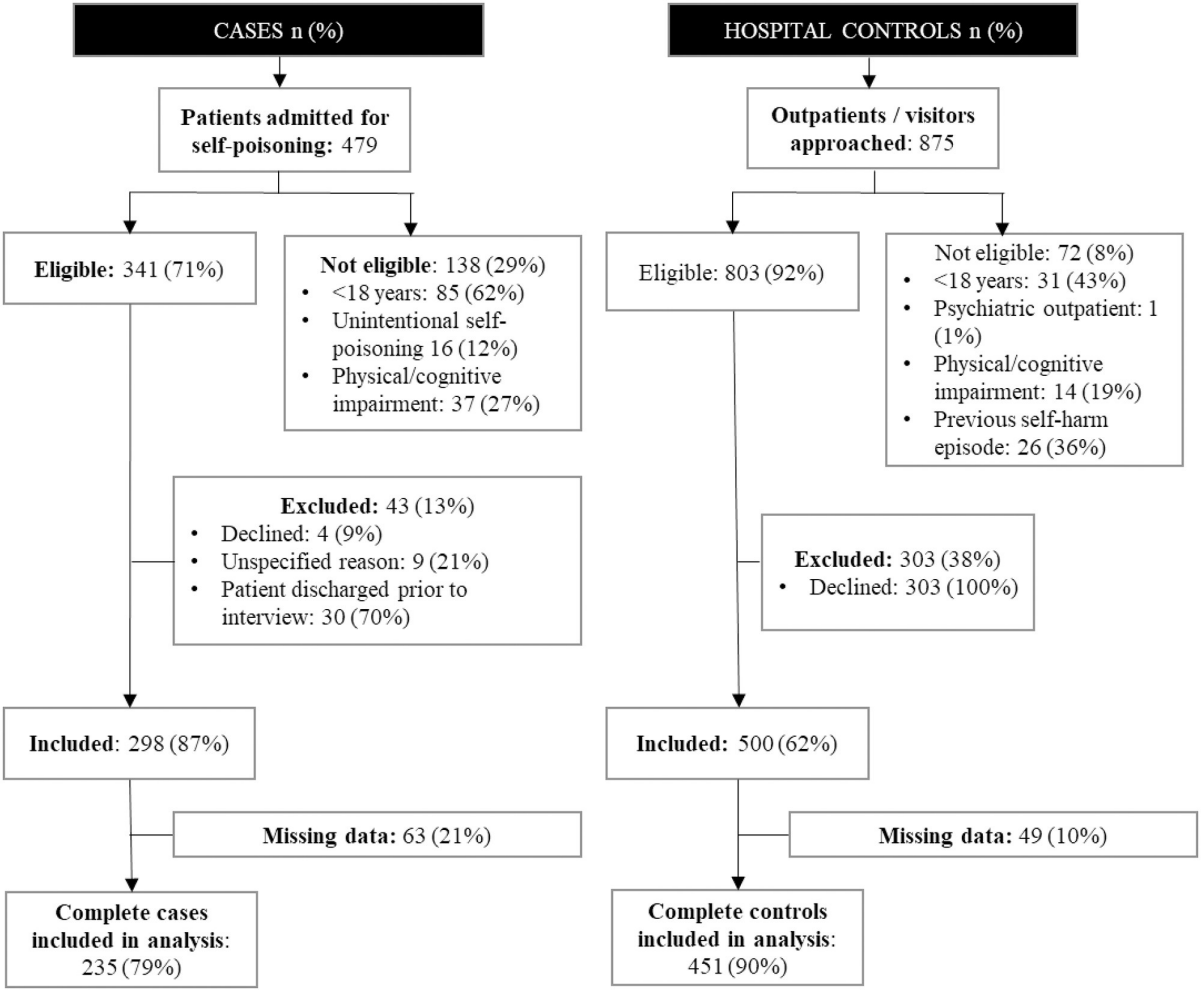
Participant recruitment for cases, and hospital controls.

A total of 341 cases and 803 hospital controls were eligible to be included in the study, while 29% of patients admitted for self-poisoning (cases) and 8% of hospital controls approached were not eligible to take part (Fig 2); 298 cases (87%) and 500 controls (62%) consented to take part in the study. All consenting participants were interviewed. The hospital controls included both outpatients (27%) and accompanying visitors (73%). An additional 455 (63% response rate) community controls were also interviewed. A higher proportion of cases (n = 63, 21%) had missing data compared to controls (n = 49, 10%), with the parental education variable having the most missing data (n = 98, 12%). A total of 235 cases and hospital 451 controls had complete data and were used in the primary analysis.

There were more female (cases n = 134; controls n = 269) than male (cases n = 101; controls = 182) participants, with a median age of 25 years (IQI 20–34) for cases and 26 years (IQI 21–36) for controls. Being non-Sinhala, non-Buddhist, and having a lower childhood socioeconomic position was associated with an increased risk of self-poisoning (Table 1). Being single reduced risk of self-poisoning compared to being married and being divorced/separated/widowed was associated with a fourfold increased risk (95% CI 1.5, 13.4). Lower levels of education, lower levels of asset ownership, harmful/hazardous drinking, moderate/severe depression and lower levels of social capital were associated with increased risk of self-poisoning; and having a young child (≤11-year-old) increased the risk of self-poisoning by 60% (Table 1).

**Table 1:** Characteristics of cases and hospital controls for key variables.

	Cases n = 235	Hospital Controls n = 451	OR	95% CI	
**Key confounders n(%)**					
Non-Sinhala ethnicity (vs Sinhala)	50 (21.3)	36 (8.0)	3.04	1.90, 4.84	<0.001
Non-Buddhist religion (vs Buddhist)	57 (24.3)	44 (9.8)	2.88	1.87, 4.45	<0.001
Childhood SEP (parent(s) highest education)					
Passed A/L or completed university/postgraduate qualifications	61 (26.0)	158 (35.0)	*reference*		
Passed O/L	54 (23.0)	137 (30.4)	1.09	0.71, 1.69	0.69
completed between grades 1–10	103 (43.8)	149 (33.0)	2.29	1.51, 3.46	0.001
No schooling	17 (7.2)	7 (1.6)	10.36	3.84, 27.94	<0.001
**Additional key factors**					
Marital status n(%)					
Married/Living with partner	122 (51.9)	204 (45.2)	*reference*		
Single	102 (43.4)	241 (53.4)	0.40	0.26, 0.60	<0.001
Divorced, separated, widowed	11 (4.7)	6 (1.3)	4.46	1.48, 13.44	0.01
Occupation n(%)					
Full time	101 (43.0)	145 (32.2)	*reference*		
Part time	4 (1.7)	6 (1.3)	0.92	0.25, 3.38	0.90
Temporary/Casual	9 (3.8)	19 (4.2)	0.63	0.27, 1.46	0.28
Self employed	5 (2.1)	13 (2.9)	0.61	0.21, 1.77	0.36
Housewife/retired	39 (16.6)	78 (17.3)	0.78	0.46, 1.33	0.36
Unemployed	77 (32.8)	190 (42.1)	0.46	0.30, 0.70	<0.001
Education n(%)					
Passed A/L or completed university/postgraduate qualifications	60 (25.5)	240 (53.2)	*reference*		
Passed O/L	79 (33.6)	124 (27.5)	2.50	1.67, 3.73	<0.001
completed between grades 1–10, or no schooling	96 (40.9)	87 (19.3)	5.16	3.37, 7.91	<0.001
Household assets n(%)					
car, tractor, bus	43 (18.3)	103 (22.8)	*reference*		
motorbike,3 wheeler	82 (34.9)	184 (40.8)	1.05	0.68, 1.64	0.82
no vehicle	110 (46.8)	164 (36.4)	1.59	1.03, 2.45	0.04
Experienced domestic violence n(%)	107 (45.5)	80 (17.7)	3.88	2.72, 5.53	<0.001
Knows someone else who has self-harmed n(%)	62 (26.4)	110 (24.4)	1.07	0.74, 1.53	0.74
Suffer from any other chronic illness or disability n(%)	44 (18.7)	71 (15.7)	1.26	0.83, 1.91	0.27
Does not have child age 11 or under n(%)	163 (69.4)	348 (77.2)	0.62	0.43, 0.89	0.01
Harmful or hazardous drinking (AUDIT≥8) n(%)	52 (22.1)	51 (11.3)	2.80	1.70, 4.63	<0.001
Moderate to severe depression (PHQ-9≥10) n(%)	110 (46.8)	73 (16.2)	4.52	3.16, 6.48	<0.001
Number of people in household mean(SD)	2.2 (0.9)	2.1 (0.7)	1.05	0.86, 1.29	0.61
Social capital (higher score, lower cohesion) mean(SD)	4.1 (3.8)	2.3 (2.7)	1.19	1.13, 1.25	<0.001

### Associations between ACE and self-poisoning

Cases were 2.5 times (95% CI 1.8, 3.6) more likely to report an ACE than controls and had higher ACE scores (Table 2 –model 2). Physical abuse (OR 4.7, 95% CI 1.2, 18.6), emotional abuse (OR 3.7, 95% CI 1.3, 10.1), living with someone who was mentally ill or suicidal (OR 2.1, 95% CI 1.2, 3.4), witnessing violence against household members (OR 2.2, 95% CI 1.4, 3.4), experience of parental death/separation/divorce (OR 3.1, 95% CI 2.0, 4.9), emotional neglect (OR 3.7, 95% CI 2.3, 6.0), community (OR 2.0, 95% CI 1.2, 3.2) and collective violence (OR 1.5, 95% CI 1.0, 2.2) experienced during childhood were all associated with an increased risk of self-poisoning (Table 2). Models adjusting for potentially confounding/mediating variables are presented in S1 Table. When stratified by sex, the associations between ACE and self-poisoning were similar (presence of any ACE: male OR 2.3 95% CI 1.3, 4.0; female OR 2.7 95% CI 1.7, 4.3) and we found no statistical evidence for an interaction between ACE frequency score (p-value = 0.76), or presence of any ACE (p-value = 0.92) and participant sex (Table 3).

**Table 2:** Adjusted associations of adverse childhood experiences and hospital presentation for self-poisoning in adulthood.

	Cases n = 235	Hospital controls n = 451	Model 1	Model 2
			OR (95% CI)	OR (95% CI)
Overall				
Presence of any ACE n(%)	178 (75.7)	254 (56.3)	2.41 (1.69, 3.44)	2.51 (1.75, 3.61)
ACE frequency score mean(SD)	1.9 (1.8)	1.1 (1.3)	1.36 (1.23, 1.51)	1.38 (1.23, 1.53)
ACE binary score mean(SD)	4.2 (2.1)	3.6 (1.9)	1.17 (1.08, 1.27)	1.18 (1.09, 1.28)
Sub categories n(%)				
Physical abuse	7 (3.0)	3 (0.7)	4.83 (1.23, 19.0)	4.72 (1.17, 18.95)
Emotional abuse	12 (5.1)	6 (1.3)	3.73 (1.37, 10.12)	3.65 (1.33, 10.07)
Contact sexual abuse	26 (11.1)	40 (8.9)	1.29 (0.76, 2.18)	1.45 (0.85, 2.47)
Substance abuser in the household	48 (20.4)	84 (18.6)	1.10 (0.74, 1.64)	1.08 (0.72, 1.63)
Incarcerated household member	20 (8.5)	27 (6.0)	1.43 (0.78, 2.61)	1.36 (0.73, 2.52)
Living with household members who were mentally ill or suicidal	35 (14.9)	35 (7.8)	2.04 (1.24, 3.36)	2.06 (1.24, 3.43)
Violence against household members	48 (20.4)	48 (10.6)	2.10 (1.35, 3.25)	2.17 (1.39, 3.38)
Parental death, separation or divorce	59 (25.1)	44 (9.8)	3.13 (2.04, 4.82)	3.12 (2.01, 4.85)
Emotional neglect	53 (22.6)	34 (7.5)	3.75 (2.34, 6.03)	3.67 (2.26, 5.97)
Physical neglect	19 (8.1)	19 (4.2)	1.94 (1.00, 3.75)	1.87 (0.95, 3.65)
Bullying	10 (4.3)	18 (4.0)	1.03 (0.46, 2.27)	0.99 (0.44, 2.24)
Community violence	38 (16.2)	42 (9.3)	1.90 (1.18, 3.04)	1.97 (1.22, 3.20)
Collective violence	61 (26.0)	90 (20.0)	1.37 (0.94, 2.00)	1.50 (1.02, 2.20)

Model 1 –adjusted for age and sex

Model 2 –adjusted for age, sex, ethnicity, religion

**Table 3:** Sex stratified adjusted associations of adverse childhood experiences and hospital presentation for self-poisoning in adulthood.

	Male			Female		
	Cases n = 101	Hospital controls n = 182	OR* (95% CI)	Cases n = 134	Hospital controls n = 269	OR* (95% CI)
Overall						
Presence of any ACE n(%)	80 (79.2)	116 (63.7)	2.26 (1.27, 4.04)	98 (73.1)	138 (51.3)	2.65 (1.65, 4.26)
ACE frequency score mean(SD)	2.1 (1.9)	1.3 (1.4)	1.37 (1.17, 1.60)	1.6 (1.7)	1.0 (1.2)	1.36 (1.16, 1.58)
ACE binary score mean(SD)	4.6 (2.3)	3.9 (1.9)	1.18 (1.05, 1.33)	3.9 (1.9)	3.3 (1.8)	1.16 (1.04, 1.31)
Sub categories n(%)						
Physical abuse	2 (2)	2 (1.1)	1.54 (0.19, 12.46)	5 (3.7)	1 (0.4)	9.75 (1.02, 92.96)
Emotional abuse	7 (6.9)	4 (2.2)	3.08 (0.86, 11.07)	5 (3.7)	2 (0.7)	4.37 (0.77, 24.71)
Contact sexual abuse	15 (14.9)	23 (12.6)	1.42 (0.69, 2.93)	11 (8.2)	17 (6.3)	1.25 (0.55, 2.84)
Substance abuser in the household	29 (28.7)	34 (18.7)	1.71 (0.96, 3.04)	19 (14.2)	50 (18.6)	0.70 (0.38, 1.28)
Incarcerated household member	11 (10.9)	11 (6.0)	1.70 (0.69, 4.16)	9 (6.7)	16 (5.9)	1.02 (0.42, 2.48)
Living with household members who were mentally ill or suicidal	13 (12.9)	13 (7.1)	2.08 (0.92, 4.72)	22 (16.4)	22 (8.2)	1.91 (0.98, 3.71)
Violence against household members	17 (16.8)	22 (12.1)	1.48 (0.74, 2.97)	31 (23.1)	26 (9.7)	2.79 (1.54, 5.05)
Parental death, separation or divorce	24 (23.8)	18 (9.9)	2.80 (1.42, 5.50)	35 (26.1)	26 (9.7)	3.52 (1.95, 6.36)
Emotional neglect	26 (25.7)	23 (12.6)	2.29 (1.21, 4.35)	27 (20.1)	11 (4.1)	6.88 (3.14, 15.1)
Physical neglect	9 (8.9)	9 (4.9)	1.68 (0.62, 4.51)	10 (7.5)	10 (3.7)	1.85 (0.71, 4.84)
Bullying	8 (7.9)	8 (4.4)	1.85 (0.67, 5.14)	2 (1.5)	10 (3.7)	0.36 (0.07, 1.80)
Community violence	22 (21.8)	19 (10.4)	2.47 (1.25, 4.88)	16 (11.9)	23 (8.6)	1.51 (0.75, 3.05)
Collective violence	33 (32.7)	44 (24.2)	1.59 (0.92, 2.76)	28 (20.9)	46 (17.1)	1.34 (0.78, 2.32)

* Model 2 –adjusted for age, sex, ethnicity, religion

#### Sensitivity analyses

In the analysis using all available data (i.e., including all cases and controls) we found very similar associations between ACE score and self-poisoning to the initial models (data not shown). We repeated the analysis using the ACE binary score (Table 2) and whilst the associations were weaker in these models, the overall findings remain unaltered. S2 and S3 Tables show demographic differences between the hospital and community control groups and model results for the community control group analysis, which were consistent with those using the hospital control group.

## Discussion

The results of this study show that childhood adversity is associated with an elevated risk of self-poisoning, with point estimates for increase in risk/odds associated with the different types of ACE ranging between one to five. Physical abuse, emotional abuse, living with someone who was mentally ill or suicidal, witnessing violence against household members, experience of parental death/separation/divorce, emotional neglect, community and collective violence experienced during childhood were all associated with an increased risk of self-poisoning, in line with our hypothesis.

### Comparison to previous studies

The findings of this study are consistent with a recent meta-analysis (2019) of 68 studies, none of which came from Asia, which found that childhood physical (OR 2.5 95% CI 2.1, 3.0) and emotional (OR 2.5 95% 1.6, 3.8) abuse were associated with an increased risk of suicide attempts [25]. The review also reported an elevated risk of suicide attempts in individuals who were sexually abused in childhood (OR 3.2 95% CI 2.8, 3.7). We did not find any statistical evidence of an association in this study with sexual abuse. The inconsistent findings may be due to differential associations in different cultures, or factors such as stigma surrounding sexual abuse which may have led to reluctance to disclose and underreporting of this adversity. More studies from South Asian countries are needed to assess this further and explore if this finding is replicable.

We found that the risk of self-poisoning was elevated in individuals who experienced parental death/separation/divorce during childhood, similar to findings from other countries [26, 27]. Previous studies have found that bereavement of a parent due to external causes (e.g., suicide) was associated with a higher degree of self-harm risk in adulthood, compared to losing a parent due to natural causes [28–30]. The effect estimates from this study were consistent with parental loss due to external rather than natural causes; however we did not have data on the causes of parental death in the current study. Exposure to community violence has also been shown to be associated with increased suicidal behaviour in HIC, and is also observed in this analysis [30].

The levels of sexual abuse reported in the control group were consistent with previously reported rates of abuse in a large cross-sectional survey (10.8% 95% CI 9.5, 12.1) [31]. However, rates of emotional abuse, physical abuse and witnessing violence against a household member were lower in this study. In our study we used a severity threshold to identify levels of abuse, which differs from the previous survey, which may have influenced the finding of lower levels. The face-to-face interviews may also have played a role, in that participants may have been reluctant to divulge sensitive information in an interview compared to an anonymous survey, even though every effort was made to minimize this possibility by having trained interviewers and conducting the interviews confidentially.

Our finding that having a lower level of education, being divorced or separated, and having fewer assets is associated with increased risk of self-poisoning is in keeping with previous findings [32]. Having a young child below the age of 11 years also increased the risk of self- poisoning, which differs from evidence from high income countries which finds that women are less likely to die by suicide if they have young children [33]. In our study, being married compared to being single, increased the risk of self-poisoning–which is in contrast to previous findings from mostly high income countries, which has described being unmarried as a risk factor for suicidal behaviour [34]. However other South Asian studies have identified marriage as being associated with increased risk of self-harm [35]. These findings may reflect the complex problems faced by lower socio-economic groups in South Asian countries; where marriage and childbearing, especially if it occurs at a young age in the context of socio-economic difficulties, may lead to higher risk of interpersonal conflict and more challenges being faced by young females with children.

#### Possible mechanisms

Childhood adversity may lead to adult suicidal behaviour via multiple pathways—exposure to childhood abuse has been shown to be associated with increased risk of adult depression in HIC, which is associated with increased risk of self-harm and suicidal behaviour [36–39]. Whilst the association between depression and suicidal behaviour is less consistently observed in LMIC in the general population, it may be that individuals who experience childhood adversity are at higher risk of developing depression as adults and engaging in suicidal behaviour [40, 41]. In keeping with this, in this study we observed that cases with self-poisoning were more likely to screen positive for depression, and adjusting for current depression reduced the association observed between ACE and self-poisoning. Adversity can have physiological impacts on individuals through biological embedding, with repeated activation of the ‘fight or flight’ response causing lasting changes in hypothalamic-pituitary-adrenal (HPA) axis functioning, which in turn impacts on multiple biological systems such as inflammation [42]. This may result in altered levels of neurotransmitters such as dopamine and serotonin, implicated in the aetiology of suicidal behaviour [42].

Childhood adversities do not occur in isolation, and previous work has shown that exposure to childhood abuse often co-occurs with other indicators of household dysfunction such as domestic violence, parental unemployment, substance misuse and poverty [43, 44]. Exposure to both childhood abuse and growing up in a household with domestic violence has a ‘double whammy’ effect, in causing a greater risk of psychological problems in adulthood [43]. As adults they are also likely to be more vulnerable to psychological problems, and externalizing behaviours such as suicidal behaviours [43]. Furthermore, as adults they are more likely to have increased emotional dysregulation and poor attachment patterns, and are vulnerable to further victimization in adult relationships, all of which may contribute towards increased risk of suicidal behaviours [43].

Exposure to community or collective violence can lead directly or indirectly to self-harm and suicide, possibly through increasing mental distress, reduced social cohesion, reducing sensitivity to violence, or by increased perception that violence is a solution. Therefore self-harm may be more cognitively available to those exposed to such violence [31, 43]. Qualitative research is needed to elucidate the possible mechanisms by which community/collective violence are associated with increased self-harm.

### Implications for practice

The results of this study show that in Sri Lanka, childhood adversity is associated with increased risk of self-poisoning. In Sri Lanka, similar to the rest of the world, minimization of self-poisoning is a complex task that would require multiple interventions at different levels. International evidence suggests that in the face of childhood adversity, the child having a secure attachment with at least one stable adult figure, or having a close connection with school could act as protective factors [31, 43]. This may be helpful in LMIC such as Sri Lanka, and the effectiveness of this recommendation will need to be explored further. In this study only a minority of participants reported no schooling (0.6%), suggesting that in Sri Lanka school-based interventions focused on identifying and supporting children experiencing abuse, neglect, and/or violence in the home (i.e., maltreatment) could be a good target for prevention. This could be done, for example, by increasing awareness among schoolteachers about the effects of exposure to adversity such as domestic violence in childhood, and provision of trained counsellors in schools. To achieve this the links between existing social, educational and health care systems should be strengthened, so schools could refer on to appropriate professionals and families and children can get ongoing support. Furthermore, given that schools have close ties with families, they are likely to be made aware of changes to the home environment (i.e., parental death, separation, divorce) and be in a position to provide support or refer to relevant services. LMIC, however, face unique challenges in this regard–there is an extreme emphasis on academic performance of students, almost to the exclusion of all else, and any intervention that is not directly aimed at increasing academic performance is likely to be perceived as a waste of time or increased burden, both by teachers and students. At the same time, teachers are overburdened, and each class has a large number of students, which prevents individual attention. Thus, any school-based strategies need to be adapted to the local environment in innovative ways, to ensure feasibility and sustainability.

Parents in Sri Lanka often underestimate the impact on children, of witnessing or experiencing domestic violence; women in particular may tolerate domestic violence for the “sake of family unity” [45]. Increased awareness among parents, both mothers and fathers, about how growing up in a household with domestic violence can impact on their children, and provision of links to support, are avenues to explore. The work of the National Child Protection Authority of Sri Lanka, and the recent establishment of the Adolescent and Youth Health Unit at the Family Health Bureau, of the Ministry of Health of Sri Lanka, indicates that this area has already been identified as a need, and maybe useful to spearhead such awareness programs and support in the community. Establishment of community support groups are also worth exploring. Healthcare professionals need to be made more aware of the potential health adversities of domestic violence. Development of mental health services has been a focus in recent years in Sri Lanka, and this needs to be an ongoing priority, together with measures to improve mental health literacy within the community.

### Strengths and limitations

Strengths of this study are the large sample size, and the high response rate of participants. Furthermore, locally validated international tools were used in the assessment of psychiatric morbidity.

Our study did not include those who attempted suicide by methods other than self-poisoning, and this may have limited the generalizability of our findings. However, in Sri Lanka the most common method of (non-fatal) self-harm is by self-poisoning. Given the retrospective nature of the study, we were not able to infer causality between associations. Selection bias may have also been introduced through using hospital-based controls; however when hospital controls were compared with community controls we found no significant difference. We were also not able to screen for degree of suicidal intent, or impulse control disorders, which is a limitation.

The ACE questionnaire used to measure childhood adversity, and the HARK questionnaire have not been validated for use in Sri Lanka, which is a limitation, but we translated, back translated and adapted these for the local context prior to use. Certain types of adversity, such as socio-economic status or poor academic performance in childhood, are not explored via the ACE. However we inquired about childhood socio-economic status separately. The ACE-IQ asks about whether or not an individual experienced a certain ACE during the first 18 years of life. For certain ACE (e.g. parental death), the experience may have differing impacts depending on the age of the child. Given the questionnaire burden of the current study we were unable to explore this and further research will be needed to investigate this in detail. Due to time restrictions we did not explicitly ask about a family history of suicide, which is a limitation. The retrospective nature of data collection regarding the experience of adversity may also have influenced our findings. We were also not able to formally explore the mechanisms which underlie the association between ACE and self-poisoning.

## Conclusions

We found that exposure to childhood adversity is associated with increased likelihood of self-poisoning. Efforts to reduce the number of children who experience adversity is a key priority in the prevention of suicide and self-harm. We recommend school and community-based interventions focused on increasing awareness of the impacts of childhood adversity, identifying and supporting children experiencing adversity. We also strongly recommend increasing awareness among families and in the community about how childhood adversity can have both short and long-term negative effects on children, and exploring innovative ways to support such families; domestic violence, and alcohol related family problems are challenging but important areas in this regard. Further research should explore factors that may promote positive outcomes in the face of childhood adversity. Locally suited programs to help young people, and families, identify and develop adaptive coping mechanisms for dealing with interpersonal conflict and acute emotional distress, is also worth exploring in future research [46].

## Supporting information

S1 Table Model 3—Adjusted for age, sex, ethnicity, religion, marital status, occupation, education, household assets, domestic violence, know someone who has self-harmed, chronic illness/disability, child ≤11 years, alcohol misuse, depression, household size, social capital, parental education. (DOCX)

S2 Table Comparison between hospital and community controls for key confounders and factors. (DOCX)

S3 Table Adjusted associations of adverse childhood experiences and hospital presentation for self-poisoning in adulthood. (DOCX)
