# Correction: Childhood adversity and self-poisoning: A hospital case control study in Sri Lanka

**DOI:** 10.1371/journal.pone.0251223

**Published:** 2021-04-29

**Authors:** Thilini Rajapakse, Abigail Emma Russell, Judi Kidger, Piumee Bandara, José A. López-López, Lalith Senarathna, Chris Metcalfe, David Gunnell, Duleeka Knipe

In Fig 1, the term self-harm should be self-poisoning. In Fig 2 under the flowchart for cases, the number of patients admitted for self-poisoning should be 481 and the not eligible number should be 140. Please see the correct Figs 1 and 2 here.

**Fig 1:**
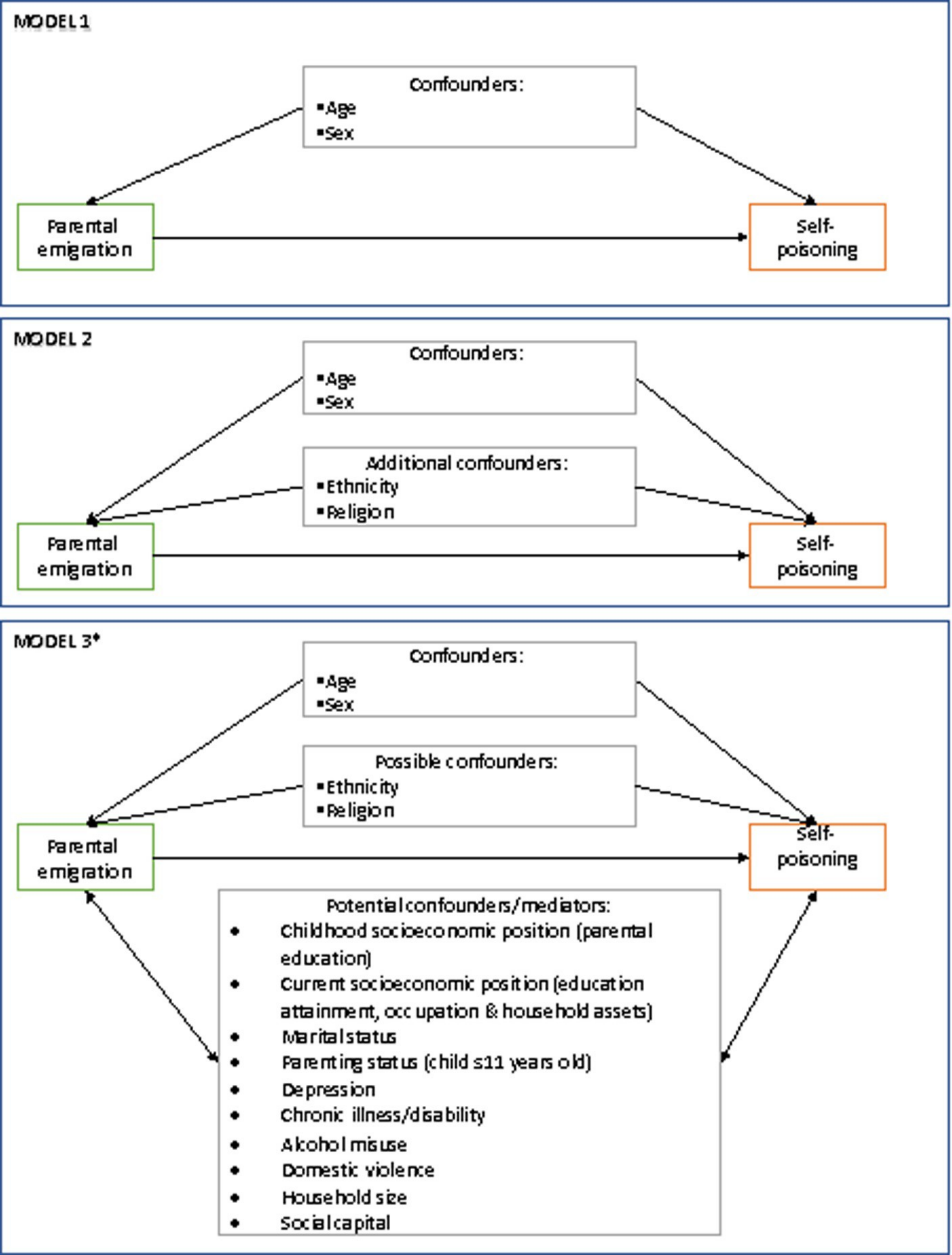
Graphical presentation of the conceptual models used to inform statistical analysis.

**Fig 2:**
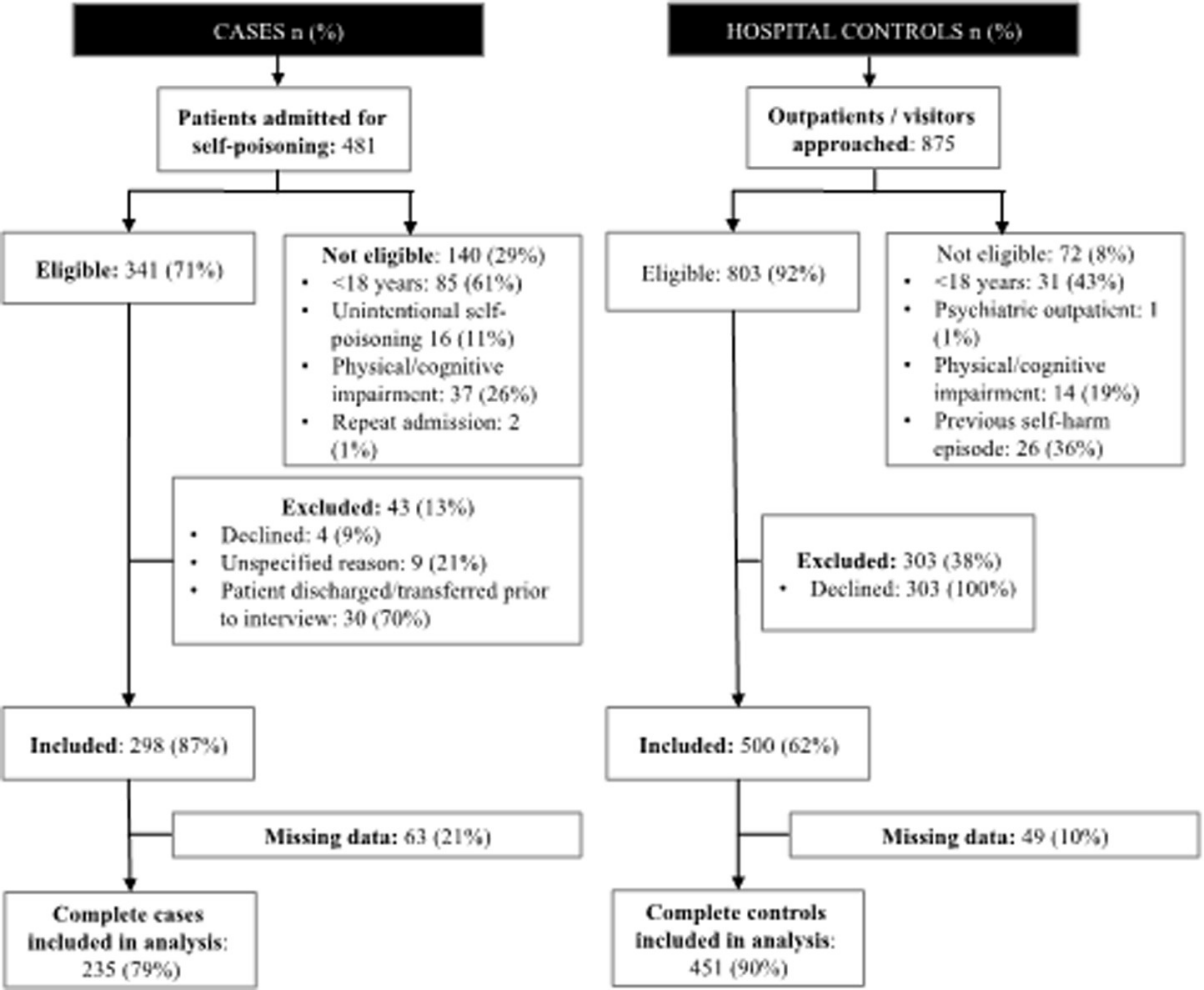
Participant recruitment for cases, and hospital controls.
