# Supplementary material for: Childhood adversity and self-poisoning: A hospital case control study in Sri Lanka
Source: PLoS One. 2020 Nov 19;15(11):e0242437. doi: 10.1371/journal.pone.0242437 (PMC7676676; doi:10.1371/journal.pone.0242437)
Supplement: S1 Table — (DOCX) [file pone.0242437.s001.docx]

**S1 Table. Model 3 – adjusted for age, sex, ethnicity, religion, marital status, occupation, education, household assets, domestic violence,**

**know someone who has self-harmed, chronic illness/disability, child ≤11 years, alcohol misuse, depression, household size,**

**social capital, parental education**

|  |  | OR (95% CI) |
| --- | --- | --- |
|  |  | Model 3 |
| Overall | |  |
|  | Presence of any ACE n(%) | 1.17 (0.75, 1.81) |
|  | ACE frequency score mean(SD) | 0.99 (0.86, 1.15) |
|  | ACE binary score mean(SD) | 0.92 (0.82, 1.03) |
| Sub categories n(%) | |  |
|  | Physical abuse | 0.96 (0.20, 4.6) |
|  | Emotional abuse | 0.94 (0.29, 3.07) |
|  | Contact sexual abuse | 1.33 (0.68, 2.60) |
|  | Substance abuser in the household | 0.48 (0.29, 0.82) |
|  | Incarcerated household member | 0.46 (0.21, 1.00) |
|  | Living with household members who were mentally ill or suicidal | 1.30 (0.69, 2.45) |
|  | Violence against household members | 0.85 (0.49, 1.49) |
|  | Parental death, separation or divorce | 2.12 (1.24, 3.60) |
|  | Emotional neglect | 1.82 (1.02, 3.27) |
|  | Physical neglect | 0.94 (0.42, 2.09) |
|  | Bullying | 0.42 (0.15, 1.15) |
|  | Community violence | 1.22 (0.68, 2.21) |
|  | Collective violence | 0.84 (0.52, 1.35) |
