# Supplementary material for: Childhood adversity and self-poisoning: A hospital case control study in Sri Lanka
Source: PLoS One. 2020 Nov 19;15(11):e0242437. doi: 10.1371/journal.pone.0242437 (PMC7676676; doi:10.1371/journal.pone.0242437)
Supplement: S2 Table — (DOCX) [file pone.0242437.s002.docx]

**S2 Table – Comparison between hospital and community controls for key confounders and factors**

|  |  | **Hospital controls* n=500** | **Community controls* n=455** |
| --- | --- | --- | --- |
| **Key confounders n(%)** | |  |  |
| Non-Sinhala ethnicity | | 44 (8.8) | 63 (13.8) |
| Non-Buddhist religion | | 52 (10.4) | 76 (16.7) |
| Childhood SEP (parent(s) highest education) | |  |  |
|  | Passed A/L or completed university/postgraduate qualifications | 160 (32) | 140 (30.8) |
|  | Passed O/L | 140 (28) | 116 (25.5) |
|  | completed between grades 1-10 | 154 (30.8) | 150 (33.0) |
|  | No schooling | 7 (1.4) | 6 (1.3) |
| **Additional key factors** | |  |  |
| Marital status n(%) | |  |  |
|  | Married/Living with partner | 234 (46.8) | 252 (55.4) |
|  | Single | 257 (51.4) | 188 (41.3) |
|  | Divorced, separated, widowed | 9 (1.8) | 15 (3.3) |
| Occupation n(%) | |  |  |
|  | Full time | 160 (32.0) | 123 (27.0) |
|  | Part time | 6 (1.2) | 10 (2.2) |
|  | Temporary/Casual | 24 (4.8) | 10 (2.2) |
|  | Self employed | 14 (2.8) | 19 (4.2) |
|  | Housewife/retired | 91 (18.2) | 114 (25.1) |
|  | Unemployed | 205 (41.0) | 179 (39.3) |
| Education n(%) | |  |  |
|  | Passed A/L or completed university/postgraduate qualifications | 251 (50.2) | 225 (49.5) |
|  | Passed O/L | 134 (26.8) | 126 (27.7) |
|  | completed between grades 1-10, or no schooling | 115 (23.0) | 104 (22.9) |
| Household assets n(%) | |  |  |
|  | car, tractor, bus | 108 (21.6) | 111 (24.4) |
|  | motorbike,3 wheeler | 204 (40.8) | 162 (35.6) |
|  | no vehicle | 188 (37.6) | 182 (40.0) |
| Experienced domestic violence n(%) | | 91 (18.2) | 72 (15.8) |
| Knows someone else who has self-harmed n(%) | | 115 (23.0) | 84 (18.5) |
| Suffer from any other chronic illness or disability n(%) | | 77 (15.4) | 66 (14.5) |
| Does not have child age 11 or under n(%) | | 385 (77.0) | 309 (67.9) |
| Harmful or hazardous drinking (AUDIT≥8) n(%) | | 56 (11.2) | 55 (12.1) |
| Moderate to severe depression (PHQ-9≥10) n(%) | | 80 (16.0) | 51 (11.2) |
| Number of people in household mean(SD) | | 2.1 (0.8) | 2.2 (0.8) |
| Social capital (higher score, lower cohesion) mean(SD) | | 2.3 (2.7) | 1.9 (2.7) |

***** Includes data for all controls regardless of missing data
