# Supplementary material for: Childhood adversity and self-poisoning: A hospital case control study in Sri Lanka
Source: PLoS One. 2020 Nov 19;15(11):e0242437. doi: 10.1371/journal.pone.0242437 (PMC7676676; doi:10.1371/journal.pone.0242437)
Supplement: S3 Table — (DOCX) [file pone.0242437.s003.docx]

**S3 Table. Adjusted associations of adverse childhood experiences and hospital presentation for self-poisoning in adulthood**

|  |  | **Cases n=235** | **Community controls n=410** | OR* (95% CI) |
| --- | --- | --- | --- | --- |
| Overall | |  |  |  |
|  | Presence of any ACE n(%) | 178 (75.7) | 235 (57.3) | 2.37 (1.65, 3.42) |
|  | ACE frequency score mean(SD) | 1.9 (1.8) | 1.3 (1.6) | 1.22 (1.11, 1.34) |
|  | ACE binary score mean(SD) | 4.2 (2.1) | 3.4 (1.8) | 1.22 (1.12, 1.33) |
| Sub categories n(%) | |  |  |  |
|  | Physical abuse | 7 (3.0) | 5 (1.2) | 2.29 (0.70, 7.52) |
|  | Emotional abuse | 12 (5.1) | 14 (3.4) | 1.22 (0.55, 2.74) |
|  | Contact sexual abuse | 26 (11.1) | 32 (7.8) | 1.47 (0.84, 2.58) |
|  | Substance abuser in the household | 48 (20.4) | 80 (19.5) | 1.04 (0.69, 1.57) |
|  | Incarcerated household member | 20 (8.5) | 20 (4.9) | 1.77 (0.92, 3.41) |
|  | Living with household members who were mentally ill or suicidal | 35 (14.9) | 35 (8.5) | 1.85 (1.11, 3.08) |
|  | Violence against household members | 48 (20.4) | 57 (13.9) | 1.57 (1.02, 2.41) |
|  | Parental death, separation or divorce | 59 (25.1) | 63 (15.4) | 1.87 (1.24, 2.82) |
|  | Emotional neglect | 53 (22.6) | 43 (10.5) | 2.69 (1.70, 4.24) |
|  | Physical neglect | 19 (8.1) | 21 (5.1) | 1.7 (0.89, 3.28) |
|  | Bullying | 10 (4.3) | 8 (2.0) | 2.05 (0.79, 5.34) |
|  | Community violence | 38 (16.2) | 74 (18) | 0.89 (0.57, 1.38) |
|  | Collective violence | 61 (26.0) | 68 (16.6) | 1.5 (1.02, 2.20) |

***** adjusted for age, sex, ethnicity, religion
